# Administrative database as a source for assessment of systemic lupus erythematosus prevalence: Estonian experience

**DOI:** 10.1186/s41927-019-0074-7

**Published:** 2019-07-25

**Authors:** Kati Otsa, Sandra Talli, Pille Harding, Eevi Parsik, Marge Esko, Anti Teepere, Marika Tammaru

**Affiliations:** 1 Department of Rheumatology, East Tallinn Central Hospital, Tallinn, Estonia; 2 Department of Rheumatology, North Estonia Medical Centre, Tallinn, Estonia; 3 Department of Rheumatology, West Tallinn Central Hospital, Tallinn, Estonia

**Keywords:** Administrative database research, Systemic lupus erythematosus, Epidemiology, Positive predictive value, Rheumatic diseases

## Abstract

**Background:**

Administrative database research is widely applied in the field of epidemiology. However, the results of the studies depend on the type of database used and the algorithms applied for case ascertainment. The optimal methodology for identifying patients with rheumatic diseases from administrative databases is yet not known. Our aim was to describe an administrative database as a source for estimation of epidemiological characteristics on an example of systemic lupus erythematosus (SLE, ICD-10 code M32) prevalence assessment in the database of the Estonian Health Insurance Fund (EHIF).

**Methods:**

Code M32 billing episodes were extracted from the EHIF database 2006–2010. For all cases where M32 was assigned by a rheumatologist less than four times during the study period, diagnosis verification process using health care providers’ (HCP) databases was applied. For M32 cases assigned by a rheumatologist four times or more, diagnoses were verified for a randomly selected sample.

**Results:**

From 677 persons with code M32 assigned in EHIF database, 404 were demonstrated having “true SLE”. The code M32 positive predictive value (PPV) for the whole EHIF database was 60%; PPV varies remarkably by specialty of a physician and repetition of the code assignment. The false positive M32 codes were predominantly initial diagnoses which were not confirmed afterwards; in many cases, a rheumatic condition other than SLE was later diagnosed.

**Conclusions:**

False positive codes due to tentative diagnoses may be characteristic for conditions with a complicated diagnosis process like SLE and need to be taken into account when performing administrative database research.

## Background

Administrative database research has been widely applied in the field of epidemiology during the recent decades. In comparison to the methods with longer traditions, e.g. cross sectional and cohort studies, the administrative database research has advantages due to a huge data amount and lower expenses. Information is often accumulated to the administrative databases without long delays, and it can be easily linked to data from other sources [1, 2]. However, database research has its inherent limitations. Created for administrative purposes, the databases usually lack detailed clinical information and the results of research depend on the type of database used and the algorithm applied for case ascertainment [2, 3].

Rheumatologic conditions are among those where administrative databases have been utilized for epidemiological research. For example, remarkable work has been done by Sasha Bernatsky and colleagues on the epidemiology of rheumatic diseases using Canadian health care databases, as well as on reviewing the validity of rheumatology-connected administrative database research [3–6]. However, the optimal methodology for identifying patients with rheumatic diseases from administrative databases is yet not known [4].

In 2017 we published the results of prevalence and incidence estimation of systemic lupus erythematosus (SLE) in Estonia based on the Estonian Health Insurance Fund (EHIF) and the Estonian Health Care Providers’ (HCP) databases [7]. The description of the organization of rheumatologic care in Estonia and utilized databases is given in details in the original paper by Otsa and colleagues. Here, we briefly summarize the previously presented information with the focus on the EHIF database.

In Estonia (population 1.3 million), rheumatological care for adults is provided by 20 rheumatologists. All practicing rheumatologists are the graduates of the Faculty of Medicine of the University of Tartu and members of the Estonian Society for Rheumatology (ESR). The curriculum includes 6 years of undergraduate studies and postgraduate specialty training for 4 years. Recertification of the rheumatologists by the ESR is mandatory after each five-year period. As a professional and scientific organization, the ESR is a member of the European League Against Rheumatism, EULAR.

Rheumatological care is concentrated in three specialized centers in the north and south of Estonia. Proportion of the private sector in rheumatology is marginal. Patients are followed in collaboration with general practitioners (GPs) involving other specialists when necessary. All clinical information is entered to the HCPs electronic databases and diagnosis codes assigned exclusively by the physicians, and the International Classification of Diseases version 10 (ICD-10) is uniformly used. The information from HCPs databases is transferred directly to the electronic billing data avoiding thereby additional steps of data extraction and re-entering.

HCPs are paid on a “fee-for-service” basis and the bills are submitted electronically without delay after the billing episode is closed. The coverage of the population by the EHIF (https://www.haigekassa.ee/en), the only organization in Estonia dealing with compulsory health insurance, is higher than 95%. The EHIF electronic database contains data on billing episodes for medical activities performed by all public and the majority of private HCPs all over the country.

EHIF database has about 200 inbuilt real time data quality checks for incoming bills. The erroneous bills are automatically transferred back to the HCPs for correction and resubmission. EHIF database utilizes ICD-10 for diagnoses coding.

In the current paper we characterize the administrative (EHIF) database as a source of assessment of SLE prevalence. The particular aims of our study were:

1. To assess the positive predictive value of ICD-10 codes M32 in the EHIF database as a whole and by diagnosis assignment category;
2. To describe the reasons for erroneous ICD-10 codes M32 by different assignment categories.

## Methods

All ICD-10 code M32 (systemic lupus erythematosus) billing episodes of individuals 20 years of age and older were extracted from the EHIF database 2006–2010. The requested variables included: billing date, person’s Estonian unique identification number, sex and birth date, and specialization and institution of the HCP. Using the identification number, the data were transformed to a person-based form.

The individuals were divided into six M32 assignment categories based on the number of billing episodes and specialization of the HCP: code M32 recorded only by a GP; only by a specialist other than a rheumatologist; by a rheumatologist one time, by a rheumatologist two times; by a rheumatologist three times, and by a rheumatologist four or more times during the study period. The categorization of patients according to the repetition of diagnosis assignment was underlaid by a presumption of higher probability of correctness of diagnoses assigned more frequently by specialists. The decision to group together the repetitions of code assignment four times or more, was at this point arbitrary and based on clinical experience of the researchers. However, we remained flexible to change the categorization principles if the implications for the other preferences would have appeared during the study.

All cases of the first five categories and a randomly selected 20% sample of M32 cases assigned by a rheumatologist four times or more were subjected to a process of diagnoses’ verification. For identification of the persons and linking different data sources, the Estonian unique identification number was used. A standard data recording form, based on the revised 1982 American College of Rheumatology (ACR) classification criteria was utilized to collect the data from different available sources in order to verify or disprove the SLE diagnosis [8]. The GPs of the patients were interviewed by mail and phone, and the HCPs’ electronic databases were searched. Fulfillment of at least four ACR criteria together with other supportive clinical information, reviewed by experienced rheumatologists (KO, EP, ME), was required for a verified SLE diagnosis. The contacted GPs were asked to indicate the reasons for assignment of the erroneous M32 diagnoses (disproved cases) by selecting one of the three predefined choices: referral diagnosis; coding error; wrong diagnosis assigned years ago and not subsequently revised. In the case of erroneous M32 diagnoses assigned by rheumatologists or other specialists, the patient’s case was followed via electronic databases by the reviewers, and, wherever possible, the reason for the code assignment was established.

The SLE cases of five categories confirmed by the verification process alongside with the confirmed (in the random sample) proportion of all cases where the code M32 was assigned by a rheumatologist four or more times (sixth assignment category) were regarded as “true positive M32 cases”. The positive predictive value (PPV) was calculated using the formula *PPV = number of true positive M32 cases/ number of all M32 assignment cases identified in EHIF*; PPV was expressed in percentages and presented together with 95% confidence interval (95% CI) for the whole sample and separately for the six assignment categories.

The reasons for erroneous M32 assignments were described, grouped, and presented separately for GPs, other specialists and rheumatologists using absolute numbers and proportions.

The detailed description of the calculation of the SLE period and point prevalence and incidence on 2006–2010 data is presented in the original paper by Otsa and colleagues. For the calculations it was assumed that there were no false negative cases (true SLE cases that had no M32 coded contacts with the health care system during a five-year period) in the population; the issue is discussed in Otsa and colleagues 2017 [7].

## Results

From 2006 to 2010 EHIF data, 9342 billing episodes with the code M32 applying to 677 people were extracted. For 48% of the cases (*n* = 326) the M32 code was applied by a rheumatologist four or more times. Due to accessibility problems (presumably not connected with the validity of the diagnosis) of the electronic HCPs’ data, 15 cases were replaced in the originally drawn 20% random sample. From the selected 65 cases all but one was confirmed as being true SLE. Regarding the rather exceptional nature of the only disproved case (discussed later), PPV of the M32 code applied by a rheumatologist four times or more was considered as being 100%. Another 351 cases underwent a verification process by which 79 (23%) diagnoses were confirmed. The GPs could not be reached in 19 cases, no electronic data was available for 6 cases of other specialists and for 9 cases of rheumatologist assignment category; in the current analysis these cases were treated as disproved cases. Overall 405 true positive M32 cases resulted in whole sample PPV 60%, PPV for assignment categories varied from 11% (M32 applied one time by a rheumatologist) to 69% (M32 applied three times by a rheumatologist) (Table 1).

**Table 1:** Positive predictive value (PPV) by M32 assignment categories and whole database

M32 assignment category	*n* (%)	SLE confirmed, *n* (%)	PPV (95%CI)
general practitioner	126 (18.6)	26 (6.4)	20.6 (13.9–28.7)
other specialist	57 (8.4)	9 (2.2)	15.8 (7.4–27.9)
1 time rheumatologist	105 (15.5)	12 (3.0)	11.4 (6.0–19.1)
2 times rheumatologist	37 (5.5)	14 (3.5)	37.8 (22.4–55.2)
3 times rheumatologist	26 (3.8)	18 (4.5)	69.2 (48.2–85.7)
4 or more times rheumatologist	326 (48.2)	325 (80.4)	100.0
Total	677 (100.0)	404 (100.0)	59.7 (55.8–63.4)

About 40% of M32 diagnoses assigned by the GPs and disproved by the verification were shown to be subsequently unconfirmed referral diagnoses to a rheumatologist. The proportion of coding errors approximated one third (Table 2).

**Table 2:** Explications for erroneous M32 diagnoses assigned by general practitioners

Explication for M32 assignment	n (%)
Referral diagnosis to a rheumatologist, diagnosis not confirmed	32 (39.5)
Coding error	26 (32.1)
M32 diagnosis erroneously assigned to a patient years ago, diagnosis reassigned automatically	15 (18.5)
No data on diagnosis assignment, currently no signs of lupus	8 (9.9)
Total	81 (100.0)

The group of other specialists, who assigned M32 to patients to whom no M32 codes were assigned by rheumatologists during 2006–2010, consisted predominantly of emergency physicians, dermatologists and nephrologists. The reasons for erroneous assignment of M32 codes by the other specialists were divided as follows: 3 cases – coding error, 9 – referral diagnosis with no subsequent alternative pathology explaining the signs and symptoms at referral, 25 – referral diagnosis with subsequent alternative pathology (including dermatological (6), other systemic connective tissue (4), musculoskeletal and renal (both 3), and miscellaneous other (9) conditions). At five cases the reason for erroneous code assignment could not be established based on available data.

More than 75% of M32 codes erroneously assigned by rheumatologists (88 cases) were primary out-patient diagnoses for referral to further examinations (Table 3). Majority of these cases were later diagnosed as other rheumatic conditions such as undifferentiated connective tissue diseases (15 cases) and rheumatoid arthritis (5 cases), followed, in descending order of frequency, by CREST-syndrome, mixed connective tissue disease and Sjögren syndrome. The three most frequent dermatological diagnoses were alopecia, photodermatitis and rosacea; other autoimmune diseases included autoimmune hepatitis and thyroiditis. The proportion of the proved coding errors was less than 3%. The only case where M32 was erroneously assigned more than four times by a rheumatologist was later diagnosed and treated as secondary syphilis. Here, the code M32 was used seven times during a period of less than a year by a rheumatologist practicing independently from the main centers.

**Table 3:** Reasons for erroneous M32 diagnoses assigned by rheumatologists

Reason for erroneous diagnosis	n (%)
Unknown	24 (20.9)
Coding error	3 (2.6)
Primary diagnosis with no subsequent alternative pathology	9 (7.8)
Primary diagnosis with subsequent alternative pathology	79 (68.7)
other systemic connective tissue	28 (24.4)
musculoskeletal/arthritis	19 (16.5)
dermatological	13 (11.3)
other autoimmune	10 (8.7)
renal	1 (0.9)
other	8 (7.0)
Total	115 (100.0)

## Discussion

Our aim was to describe an administrative database as a source for estimation of epidemiological characteristics of a rheumatic condition. For this purpose, we utilized an example of SLE prevalence assessment in the database of the Estonian Health Insurance Fund.

The EHIF database can be considered as being in a favorable position for retrieval of reliable estimates in epidemiological studies. The completeness of data is secured by homogeneity of the health care and insurance system, imposed on a small-sized population. Application of a “fee-for-service” billing principle backs complete capture of HCP activities [9]. Data transmission design, through which data entered by a physician is transferred to the billing claims without re-entering, allows for avoiding errors caused by repeated data processing by nonmedical personnel [9, 10]. Real time data transmission with inbuilt quality checks provides the researchers with cleaned up-to-date data.

As every database is created mainly for administrative purposes, the EHIF database has its limitations in regard to epidemiological research. The EHIF does not distinguish between referral and final clinical diagnoses which may bring along a considerable number of false positive diagnostic codes in the database. Moreover, in the case of conditions with as complicated a diagnostic process as SLE, the initial diagnosis may be revised as the disease evolves in the course of time [4]. The lack of detailed clinical data in the EHIF database brings along the necessity for ascertainment of diagnoses using data sources that contain information for assessment of validity of the coded diagnosis. HCP electronic databases can be utilized for this purpose. In Estonia, the search for clinical records is facilitated by a limited number of structurally similar HCP electronic database versions in use. Our choice to contact GPs by mail was driven by the intention to speed up the data collection process as GPs approached their databases simultaneously; the more time consuming procedure of reviewing of the GPs’ databases by the researchers would have yielded apparently analogous results.

Our study design matched the approach 2b described in Widdifield and colleagues [4]: patients were sampled from the administrative database (EHIF) by the presence of diagnoses codes and were classified as true cases or false positive cases by the reference standard (HCP electronic databases). This approach precludes identification of false and true negative cases and hence calculation of a database’s sensitivity and specificity. PPV, a statistic reporting the proportion of people with the code that truly has the disease, can be estimated based on the identified false and true positives. PPV is the most commonly used statistic to report code accuracy in administrative database research validation studies [9, 11]. Although PPV use is limited in some research circumstances due to its dependency on prevalence [9, 12], this characteristic of PPV should not preclude its usage for demonstration of accuracy of diagnosis code assignment in a particular predefined group during the fixed study period [13].

The proportion of false positive M32 diagnoses in the EHIF database (40%) was similar to the 43% reported by Bernatsky and colleagues in an administrative database in Nova Scotia, Canada [4]. However, the general comparison may not be of great value for inferences, hence accuracy of diagnostic code depends on several factors. Besides the purpose of the administrative database creation, the correctness of code is greatly affected by the case ascertainment algorithm in the study [3, 4]. For confirmation of a M32 diagnosis as true SLE, we used the opinion of experienced rheumatologists on the case’s fulfillment of ACR criteria as “gold standard”. Based on the revision of clinical documentation, this approach provided us with the access to data from the six-year period after the end of cases’ enrollment. It gave us the advantage to follow the patient’s progress over a longer time interval which is valuable in case of complicated diagnoses. Our decision – to regard as true SLE cases the individuals who were assigned M32 by a rheumatologist four or more times during the studied period – may have artificially to some degree decreased our estimation of the false positive percentage. Yet, our data revealed a decrease in the false positive proportion from about 90 to 60% to 30% among the cases coded M32 respectively once, twice and three times by a rheumatologist. During the verification of a random sample of the M32 diagnoses assigned four times or more, only one rather exceptional false positive case was detected. Thereby, the percentage of false positives could be assumed as being further diminished with increment of M32 assignment repetitions, finally approximating zero. Our results corroborated the earlier results of administrative database research by Bernatsky, Widdifield and colleagues demonstrating the effect of specialty of physician on the accuracy of diagnosis of rheumatic condition [4, 5]. PPV of the M32 codes assigned by GPs and specialists other than rheumatologists ranged from 15 to 20%. Among the rheumatologists’ diagnoses, the proportion of false positives decreased with an increasing number of billing episodes with PPV varying from 10 to 70% among codes assigned once and three times during the study period, respectively.

The false positive diagnoses assigned both by the GPs and other specialists were predominantly referral diagnoses which were not confirmed by a rheumatologist afterwards. Similarly, the majority (about 70%) of false positive M32 codes assigned by the rheumatologists turned out to be primary diagnoses which were not confirmed by the further examination. These results support the findings of Bernatsky and colleagues of the initial diagnoses being a major source of low PPV of administrative databases in the case of rheumatic conditions [4]. Due to the evolving nature of SLE and relying on the finding that many initial M32 diagnosis cases were later diagnosed as having other systemic connective tissue diseases, it may be argued that decreased validity caused by tentative diagnoses is and will be an intrinsic part of administrative database research of SLE epidemiology. A potentially avoidable cause of false positivity – coding error – contributed to a relatively small proportion of PPV decrease in our study among rheumatologists and other specialists. Coding errors made by the GPs occurred mostly in the cases of conditions with similar ICD codes (e.g. F32, H32, N32) and could presumably be attributed to the beginning of the study period when prescriptions were still handwritten. Regarding the digitalized prescription system, which was introduced to Estonian health care in 2010 and is used in an almost exceptional manner today, the role of coding error as a reason for false positive M32 codes can be expected to have decreased.

Although SLE and syphilis may share common clinical and laboratory features [14, 15], we would like to believe that syphilis misdiagnosed as SLE during a year by a rheumatologist is a regrettable exception. According to the Estonian Health Board (http://www.terviseamet.ee/en/information.html) there were 166 cases of early syphilis diagnosed in Estonia during 2006–2011. Remarkably there were no misdiagnosed syphilis cases among the false positive M32 diagnoses assigned by rheumatologists one, two or three times. In our opinion, this supports the decision to treat the only misdiagnosed case as a highly uncommon occurrence. The case can be used as an illustration of the importance of concentration of rheumatological care to centers with high level diagnostic possibilities and accumulation of knowledge and experience.

In our sample, the correctness of M32 code assignment did not depend on patients’ age and sex (logistic regression analysis, results not shown); these results contradicts the findings of Bernatsky and colleagues of lower sensitivity of case definitions of systemic autoimmune diseases in billing data for older individuals [4].

## Conclusions

The administrative database research, a relatively new approach in epidemiology, has some clear advantages over other more longstanding methods and may be an especially useful source to estimate the incidence and prevalence of rare diseases such as SLE [16]. Administrative database research also has its inherent limitations; the necessity for validation of diagnoses in administrative data using other data sources consumes time and resources and may be complicated by the issues of data protection legislation. Generalisability of the results of database research is affected by limitations of application of case ascertainment algorithms to administrative databases of different structure and functioning principles. We hope that the detailed description of the used database and methods provides the readers with grounds to decide on applicability of our findings in their specific context of interest.

Based on ours and other similar studies’ results we dare to put forward an assumption that in the case of the SLE there are two types of “false positive” diagnoses in administrative databases. Occurrence of the first type can be diminished to approximately zero by further enhancement and automation of data collection processes; an example of this type of “false positive” is coding error. The second type originates from the nature of SLE – a rare condition with an often unspecific onset, low predictability of development and a generally complicated diagnostic process. These characteristics brings along an inevitable period of “diagnostic hesitancy” – referral and initial diagnoses that may and may not in time result in a “true SLE” case. The probability of a diagnosis being tentative varies according to the level of a HCP due to different training, diagnostic possibilities and patients’ population seen. Unlike the type one “false positives”, those presenting the second type cannot be made to disappear by improving the data collection process and database functioning. Referral and initial diagnoses will constitute a source for “false positive” database diagnoses until there are some major developments in the SLE diagnostic process or changes in administrative databases’ structuring principles are done. The proportion of “false positives” due to tentative diagnoses in SLE administrative data can hence be assumed stable for some forthcoming time. It allows us to believe that besides being a source for one time estimation of SLE epidemiological characteristics, an administrative database of certain technical perfection can be used for monitoring changes in condition prevalence with precision sufficient for informing health care policy. A research challenge would be to create condition- and database-specific simple algorithms for estimation of diagnosis validity, and to apply them on the incoming data in order to monitor the rough trends in the condition’s prevalence. So far, the researches should keep in mind that usage of administrative data which include cases with few repetitions of M32 codes may lead to overestimation of SLE prevalence.

## Data Availability

All data generated and analysed during this study are available from the corresponding author on request.

## Abbreviations

ACR: American College of Rheumatology
CI: Confidence interval
EHIF: Estonian Health Insurance Fund
ESR: Estonian Society for Rheumatology
EULAR: European League Against Rheumatism
GP: General practitioner
HCP: Health Care Providers
ICD-10: International Classification of Diseases version 10
PPV: Positive predictive value
SLE: Systemic lupus erythematosus
